# Electromyographic Assessment of the Efficacy of Deep Dry Needling versus the Ischemic Compression Technique in Gastrocnemius of Medium-Distance Triathletes

**DOI:** 10.3390/s21092906

**Published:** 2021-04-21

**Authors:** María Benito-de-Pedro, César Calvo-Lobo, Daniel López-López, Ana Isabel Benito-de-Pedro, Carlos Romero-Morales, Marta San-Antolín, Davinia Vicente-Campos, David Rodríguez-Sanz

**Affiliations:** 1 Faculty of Nursing, Physiotherapy and Podiatry, Universidad Complutense de Madrid, 28040 Madrid, Spain; mariabenito1309@gmail.com (M.B.-d.-P.); cescalvo@ucm.es (C.C.-L.); anabenito1912@gmail.com (A.I.B.-d.-P.); davidrodriguezsanz@ucm.es (D.R.-S.); 2 Research, Health and Podiatry Group, Department of Health Sciences, Faculty of Nursing and Podiatry, Universidade da Coruña, 15403 Ferrol, Spain; daniellopez@udc.es; 3 Villaviciosa de Odón Campus, Universidad Europea de Madrid, 28670 Madrid, Spain; carlos.romero@universidadeuropea.es; 4 Facultad de Ciencias de la Salud, Universidad Francisco de Vitoria, Pozuelo de Alarcon, 28223 Madrid, Spain; Davinia.vicente@ufv.es

**Keywords:** myofascial pain syndrome, trigger points, electromyography, deep dry needling, ischemic pressure technique

## Abstract

Several studies have shown that gastrocnemius is frequently injured in triathletes. The causes of these injuries are similar to those that cause the appearance of the myofascial pain syndrome (MPS). The ischemic compression technique (ICT) and deep dry needling (DDN) are considered two of the main MPS treatment methods in latent myofascial trigger points (MTrPs). In this study superficial electromyographic (EMG) activity in lateral and medial gastrocnemius of triathletes with latent MTrPs was measured before and immediately after either DDN or ICT treatment. Taking into account superficial EMG activity of lateral and medial gastrocnemius, the immediate effectiveness in latent MTrPs of both DDN and ICT was compared. A total of 34 triathletes was randomly divided in two groups. The first and second groups (*n* = 17 in each group) underwent only one session of DDN and ICT, respectively. EMG measurement of gastrocnemius was assessed before and immediately after treatment. Statistically significant differences (*p* = 0.037) were shown for a reduction of superficial EMG measurements differences (%) of the experimental group (DDN) with respect to the intervention group (ICT) at a speed of 1 m/s immediately after both interventions, although not at speeds of 1.5 m/s or 2.5 m/s. A statistically significant linear regression prediction model was shown for EMG outcome measurement differences at V1 (speed of 1 m/s) which was only predicted for the treatment group (*R*^2^ = 0.129; β = 8.054; F = 4.734; *p* = 0.037) showing a reduction of this difference under DDN treatment. DDN administration requires experience and excellent anatomical knowledge. According to our findings immediately after treatment of latent MTrPs, DDN could be advisable for triathletes who train at a speed lower than 1 m/s, while ICT could be a more advisable technique than DDN for training or competitions at speeds greater than 1.5 m/s.

## 1. Introduction

The triceps surae complex includes both gastrocnemius and soleus muscles with attachments proximally at the posterior part of the knee and distally at the posterior part of the ankle [1], respectively. The triceps surae assists in control of the knee and ankle and thus, plays a role in all lower extremity activities including triathlon [2,3]. Currently, a triathlon (swimming, cycling, running) is a growing sport activity [4], with an exponential increase in their annual licenses [5]. Several studies have shown that the area that suffers the greatest number of injuries in this sport is the triceps surae [6,7].

Myofascial pain syndrome (MPS) is a regional pain condition associated with the presence of myofascial trigger points (MTrPs) [8]. The presence of MTrPs is considered to be the first sign of muscle overload [9]. This disorder can affect any skeletal muscle in the body and it usually accounts for 21% of orthopaedic clinic visits, 30% of general medicine visits, and around 85%–93% of patients who are referred to pain management clinics [10]. MPS usually occurs in unconditioned muscles, under high tension conditions or after direct local trauma or strains [11]. The presence of MTrPs is linked to overuse and inappropriate muscle use [8].

MTrPs which may be considered as the primary source of pain in MPS were defined as hyperirritable spots within a taut band of skeletal muscle [12]. Two different kinds of MTrPs were divided according to their classification by clinical activity: (1) latent and (2) active MTrPs [9,13]. Latent MTrPs can develop after maintaining muscle activation for long periods of time, exaggerated muscle contractions, or repeated physical activity. Latent MTrPs pain can be triggered by digital compression, stretching, and/or overload [10]. 

A very high percentage of injuries in triathlon usually occur mainly for two reasons: (1) micro-trauma of repetition or traumatisms and (2) muscular overuse [14], both of which are the main reason for the appearance of MPS [15].

Treatment of this musculoskeletal condition includes deactivation of MTrPs by procedures such as deep dry needling (DDN) or ischemic compression technique (ICT), in order to evaluate their effects on pain and functionality. DDN is considered as a safe and effective method for decreasing pain and improving function by eliciting a local twitch response (LTR) in the MTrPs [16]. Active MTrPs are associated with higher motor end plate noise than latent MTrPs, which means that latent MTrPs can display less irritability than active MTrPs and local twitch responses could be elicited with more difficulty [17]. Eliciting LTR during treatment with DDN would modulate a motor-neuron activity and disrupt the abnormal motor end plante activity [18]. The evidence suggests that this method of treatment performed by physical therapists was more effective than no treatment, sham dry needling and/or other types of treatment [19]. 

Another efficient technique for treating MTrPs is manual local ICT [20], whereby a pressure for 90 s using the thumb is applied to the MTrP [21]. ICT is considered the most common, non-invasive therapy currently used for the treatment of MTrPs [22].

Diverse studies report alterations in muscle function such as, electromyographic activity increased in the antagonist and synergists muscles of subjects with latent MTrPs [23,24], muscle fatigue increased and overload of motor units close to the latent MTrPs [25]. Moreover, latent MTrPs contribute to accelerated fatigability [26].

When performing a surface electromyography (EMG) of the muscles with MTrPs, resting activity is rarely recorded in them, but they usually show increased motor activity during contraction [10]; however, when the test is performed by means of an intramuscular needle EMG; of an unstimulated MTrP, greater spontaneous activity is observed in MTrP locations than in sites located outside of these MTrPs [27,28]. Spontaneous electrical activity (SEA) is an indication of spontaneous release of acetylcholine (ACh) at the neuromuscular junction (NMJ) [29]. This activity is characteristic of both active and latent MTrPs, which is recorded with an EMG intramuscular needle, when the muscle is at rest. The potential of the terminal motor plate in dysfunction of the extrafusal fibre of the muscle, may present two characteristics under the presence of an MTrP: (1) On one hand, a low degree of continuous electrical activity is present and represents an abnormal activity of end plate or end plate noise and (2) on the other hand, higher amplitude peaks or end plate peaks are accompanied by end plate noise of latent MTrPs [28].

Stimulation of a latent MTrPs (either by ICT or DDN) induces local pain and/or referred pain and presents a lower degree of pain in latent MTrPs than in active ones, without finding any type of end plate noise in areas of non-MTrPs [27]. 

The mode of action of the DDN is associated withs both mechanical and neurophysiological mechanisms, but still the specific mechanism of action of the DDN is not yet known exactly [30,31]. It is intended that the mechanical effects of the needle interrupt the integrity of the terminal motor plate in dysfunction [32] and alterations in the length and tension of muscle fibres [33] increase blood flow in the muscle and therefore oxygenation there in [33,34]. Kubo et al. found that DDN caused an increase in blood flow at the needling site, for 30 min starting at the moment of needling onwards [33]. Sato et al. described that the release of vasoactive substance, such as calcitonin gene-related peptide (CGRP) and substance *p* (SP) leads to vasodilatation in small vessels and increased blood flow [35].

Until now we have not found any article that measures the muscle activity in the gastrocnemius at an electromyographic level immediately after the latent PGM puncture is carried out, for this reason we have considered it relevant to carry out this investigation, in order to demonstrate whether the proven acceleration of muscle fatigue typical of latent PGM is reduced immediately after treatment with PSP and/or ICT, as measured by surface electromyography.

The aim of the study was to compare the immediate effectiveness in latent medial and lateral gastrocnemius MTrPs of both DDN and ICT assessed by superficial EMG. We hypothesized a greater improvement in EMG activity in triathletes who received DDN than in those receiving ICT.

## 2. Methods

### 2.1. Design

The current study was a secondary analysis of a single blinded clinical trial [36] that used a randomized between-group design to investigate the immediate superficial electromiographic effects of DDN treatment with a single session of this technique versus a single treatment session of ICT. Both treatments were applied to latent medial and lateral gastrocnemius MTrPs of medium-distance triathletes. The principal outcome of this clincal trial was identification of EMG activity over the latent treated MTrPs. This superficial EMG measurement was carried out for both groups of triathletes according to our previously published randomized clinical trial protocol [36]. Measurements were made in all participants by a blinded evaluator before and 5, 10, 15, 20 and 25 min after treatment session and those measurements were made 24 h after the last sport session. The study was approved by the human research committee of the Hospital Clinico San Carlos, Madrid-Spain (CEIC Hospital Clínico San Carlos 02/17), and before participation in the study all subjects signed an informed consent form. This clinical trial was prospectively registered at Clinicaltrials.gov with the number NCT03273985.

### 2.2. Participants

Participants in this study were medium distance triathletes ((1.9 km of swimming, 90 km of cycling, and 21 km of running) [4] who were recruited from the podiatric and physiotherapy clinic, Fisiofuenla s.l.p, from September to December 2017, according to a randomized sampling method. All of them had ≥3 years of experience participating in triathlons and they trained around 15 to 20 h weekly. They were selected after identifying them via a clinical exploration conducted by the principal investigator in which, the presence in gastrocnemius of latent MTrPs in the places determined by Travell and Simons [37] as the most common and symptomatic, and identification based on selection criteria were used to select the participants. All participants were evaluated by the same principal investigator who has extensive experience, at least three years, in MTrPs diagnosis and its treatment, which increased confidence in the identification of MTrPs [38]. Inclusion criteria necessary for the participation of triathletes in the study consisted of two parameters: (1) presence of a knot or hypersensitive point in the taut band of skeletal muscle and (2) presence of local or referred pain after mechanical stimulation in the superficial area of the latent MTrP. Several criteria were used to exclude the triathlete from the study: (1) Age > 75 and <18 years; (2) score ≥ 4 on the Douler Neuropathique 4 (DN4) questionnaire, which denotes lower limb neurological disorders [39]; (3) cognitive alterations according to positive results on the Pfeiffer questionnaire [40]; (4) taking anticoagulant or antiaggregant medication; (5) existence of prosthesis in the lower limb; (6) presence of systemic or local infection in the lower limb; (7) fibromyalgia, autoimmune disease, iron deficiency, or hypothyroidism; and (8) fear of needles.

### 2.3. Simple Size Calculation

A sample size calculation using the G*Power 3.1.9.2 software (G*Power^©^; University of Dusseldorf, Dusseldorf, Germany) was obtained according to the difference between two independent groups. This calculation was based on the EMG percentage (%) at a medium speed of 1.5 m/s (V2) for voluntary isometric maximum force contraction (MVC) of the latent MTrP of medial and lateral gastrocnemius muscle measured immediately after interventions. A pilot study (*n* = 12) with two groups consisting of six triathletes in the experimental group who received DDN (expressed as mean ± standard deviation [SD] of 22.96% ± 8.38%) and six triathletes in the intervention group who received ICT (35.41% ± 16.24%) [41] was initially conducted. In addition, a one-tailed hypothesis, effect size of 0.96, α error probability of 0.05, power (1-β error probability) of 0.80, and allocation ratio N_2_/N_1_ = 1 were used in order to determine the suitable sample size calculation. Therefore, a sample size of 30 triathletes was calculated with an actual power of 0.82. Finally, regarding possible 10% loss during follow-up, a total sample size of 34 triathletes with ± 17 triathletes in each of the experimental groups (DDN and ICT, respectively), was used.

### 2.4. Principal Outcome: Electromyographic Measurement

A double channel surface bipolar EMG was used to measure muscular activity in the gastrocnemius, (Verity Medical Ltd., Hans, UK), EMG ranged from 0.2 to 2000 µV root mean square (RMS prolonged) with a sensitivity of 0.1 µV RMS, accuracy of 4% of indications µV 0.3 µV at 200 Hz, and selective band filter of bandwidth 3 dB differential instrumentation amplifier.

Electrode preparation and placement of was carried out following the Surface EMG Recommendations for Non-invasive Assessment of Muscles (SENIAM) recommendations [42]. The two electrodes were placed longitudinally after careful preparation of the skin in which each muscle was cleaned with 70% alcohol, marked with and indelible marker and then shaved. The skin was shaved and scratched with fine sandpaper and then cleaned with ethyl alcohol [43]. The electrodes were secured with hypoallergenic adhesive plaster [42] and an elastic bandage was placed on them [44] in order to prevent their movement.

The location of surface electrodes was established by SENIAM protocol. According to the protocol, the first electrode should be located in external gastrocnemius near the junction line between the fibula head and heel. We placed the electrode at 1/3 of the fibula head following the line of junction [45]. The second one was located in internal gastrocnemius the electrodes were placed on the most promising area of the muscle [45]. 

Silver or silver/chloride electrodes with rectangular shape and a measure of 4 × 5 mm embedded in a round gel pad of 10 mm diameter (MedicotestGmbH, Andernach, Germany) were used and the reference ground electrode was placed in a free area of the muscle over the femur [42].

This study was divided into two parts. First, the patient was asked for a voluntary isometric maximum force contraction (MVC) [46] after receiving precise instructions on how to perform the MVC of the muscle in question. Three maximal attempts of 6 s each were performed, separated by 3 min to recover from medial and lateral gastrocnemius fatigue, and only the best performance was chosen for the statistical analysis [47]. The participants were instructed to exert maximum effort against resistance. Patients were placed in prone position, and a plantar flexion movement of the tibio-fibular talar joint, with the knee extended (a specialized therapist exerted maximum resistance on the sole of the foot) [48] was required. Patients were verbally encouraged while performing the evaluation. The purpose of this test is to allow the researcher to compare the maximum amplitudes with submaximal tasks, such as walking [44].

Certain studies with intramuscular EMG indicate that muscle with latent MTrP fatigues faster than normal muscles [26]. In 2011 Hong and Arendt Nielsen showed that reduction in mean power spectral frequency of intramuscular EMG activity occurred earlier in latent MTrPs than in normal muscle fibers [26].

Therefore, according to the SENIAM protocol, a comparison was carried out with EMG measurement on the same reference points in the affected leg [42], before and after treatment. The measurements were taken in order to evaluate muscle activity in the gastrocnemius with the superficial EMG selected for this study showing high intra and inter-examiner reliabilities (intraclass correlation coefficient from [ICC] ranging from 0.94 to 0.98). This measurement was performed in the second part of the test protocol at three different speeds on a treadmill before and after treatment (V1: 1 m/sg, V2: 1.5 m/sg, and V3: 2.5 m/sg). To determine the fastest speed that the subject could successfully complete, they were urged to begin the test on the treadmill for the lowest speed before and after treatment. If the patient could walk comfortably at this speed during the assigned 2 min period, the speed of the treadmill was increased to the next level. The study continued until maximum speed data were collected on the treadmill or until the subject could not maintain the established speed [48].

To carry out the data analysis (Software Watscope, Digital North Inc. Waterloo, ON, Canada), the first and last 5 s of the EMG study signals were selected, and the mean power frequency (FPM) was calculated in each segment [49].

### 2.5. Treatment Allocation

First, an external researcher collected the necessary subject data who participated in this study. Afterwards, the distribution of the same was carried out in both groups, DDN or ICT. This distribution was determined with the statistical and epidemiological analysis system called Epidat 4.2 (Consejería de Sanidad, Xunta de Galicia (España); Organización Panamericana de Salud (OPS-OMS); Universidad CES Colombia). Individual and numbered sheets were prepared sequentially with randomized assignment and placed in sealed opaque envelopes. A second investigator was in charge of opening the envelope. Each variable was measured five times before and after the intervention by an independent investigator.

### 2.6. Experimental Group: DDN

The experimental group of triathletes of this study received only one session of DDN for which disposable stainless-steel needles (0.3 × 50 mm, Agupunt, Madrid, Spain) were used. These needles were introduced into the MTrP after its location within the taut band [50]. The “fast in, fast out” technique was selected to carry out the DDN treatment [16]. First, the area was cleaned with alcohol, and the therapist wore sterile gloves. The needle was then held firmly between the thumb and the index finger and was directed with deepening penetration through the MTrP [51]. The needle moved up and down without exiting the skin. Finally, the DDN technique was applied for a maximum number of 8–10 insertions or up to the limit of patient tolerance [52].

### 2.7. Intervention Group: ICT 

Ischemic compression is a habitually manual pressure technique. In the present study this technique was applied to the intervention group of triathletes. In order to carry out this treatment, pressure was applied with the thumb in the latent MTrP until the pain reached its maximal tolerable level. This pressure was sustained for 1 min [53].

### 2.8. Statistical Analysis

Statistical analyses were performed by the software IBM SPSS (version 19.0, IBM Corp., Armonk, NY, USA). The Shapiro-Wilk test was performed to assess normality. Group differences were examined using, the Student’s t-test for independent variables for the variables that were adjusted to normal (*p* > 0.05). For the variables that were not adjusted to normal, the non-parametric Mann–Whitney U test (*p* < 0.05) was used. These statistical analyses were carried out according to the provided sample size calculation and following prior published secondary analyses of this randomized clinical trial [36,54]. The mean ± SD, range (minimum – maximum), mean differences and the 95% confidence intervals (CI) for each outcome measurement were calculated. Furthermore, bar graphs including 95% CI error bars were added in order to illustrate statistically significant differences for parametric data. All analyses were considered statistically significant at *p* < 0.05, with a CI of 95%.

In addition, for outcome measurements, reliability analyses were performed. Calculation of ICCs, minimum detectable changes (MDCs), standard errors of measurement (SEM) and lower and upper limits of the 95% CI were obtained. The formula SEM = SD × √(1-ICC) was used to calculate SEM values and thus measure the error range of the parameter. The MDC values were calculated to determine the change in magnitude necessary so that without being influenced by random variations or measurement errors, confidence changes are provided. SEM and MDC were analysed according to Bland and Altman. The classification of ICC values was as follows: the value was considered poor when ICC < 0.40, ICC was fair with an ICC value of 0.40–0.59. ICC was considered good with value range from 0.60 to 0.74 and ICC value ranging from 0.75–1 was categorized as excellent [55].

Finally, multivariate regression analyses were carried out in order to predict the EMG differences after treatments. Linear regression models were carried out by the stepwise selection method to predict the outcome measurements differences for EMG values at V1 (speed of 1 m/s), V2 (speed of 1.5 m/s) and V3 (speed of 2.5 m/sg) as dependent variables. Descriptive data, such as sex, height, weight, BMI and foot length, as well as treatment group (DDN = 0; ICT = 1) were included as independent variables. *R*^2^ coefficients were used to determine the quality of adjustment. Pre-stablished P_in_ and P_out_ F-probability values were set at 0.05 and 0.10, respectively.

## 3. Results

**Figure 1:**
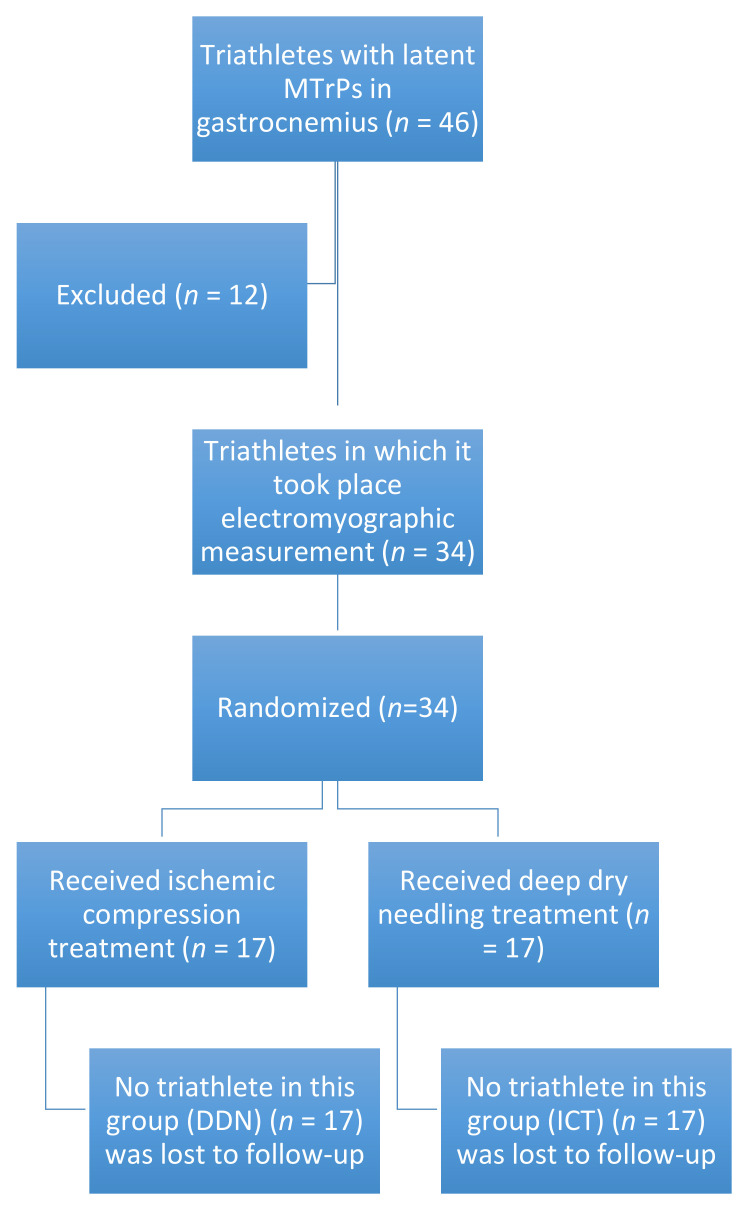
Flow diagram of patients throughout the course of the study.

A total of 46 triathletes were recruited initially to participate in this study. Of all participants, 12 participants were excluded from the study. Ten were excluded because they did not present latent MTrPs at the time of the evaluation and two because they took the medication at the time of study completion. Finally, 34 subjects (20 males and 14 females) agreed to participate in the study. None of the subjects presented any adverse effects (Figure 1).

### 3.1. Sociodemographic Characteristic by Treatment Groups.

The demographic data of the sample studied were divided by type of treatment. The experimental group was treated with DDN, and the intervention group was treated with ICT. Ages, both in the experimental and intervention groups (35.29 ± 5.39 and 33.76 ± 76 years, respectively) did not present statistically significant differences between them (*p* = 0.215). Weight did not present presented statistically significant differences between experimental and intervention groups 65.17 ± 10.71 and 69.17 ± 10.66 kg, respectively; *p* = 0.141. Height did not present statistically significant differences between both experimental and intervention groups 170.35 ± 12.94 and 174.94 ± 6.96 cm, respectively; *p* = 0.103. Body mass index (BMI) did not present statistically significant differences in the experimental and intervention groups 22.37 ± 1.92 and 22.48 ± 2.35 kg/cm^2^, respectively; *p* = 0.443. Foot length did not show any statistically significant differences between both groups (*p* = 0.421) with values in the experimental and intervention groups of 41.55 ± 3.26 and 41.35 ± 2.73 cm, respectively; These results matched the randomization of the sample when selecting for one treatment or another.

### 3.2. Electromyographic Measurement 

**Figure 2:**
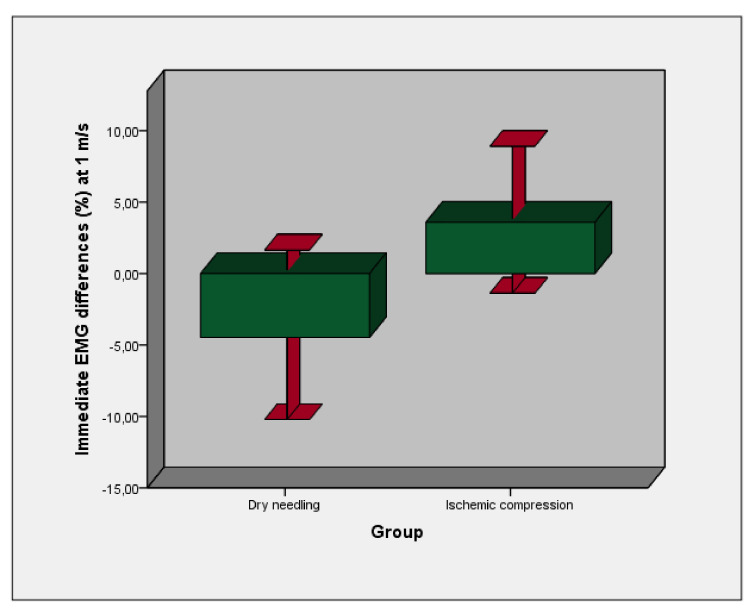
Bars graph with 95% confidence intervals (CI) error bars of immediate electromyograp- ic measurement differences after experimental (DDN) and control (ICT) interventions of muscle activity in medial or lateral gastrocnemius at a speed of 1 m/s (V1).

According to Table 1 and Table 2 and Figure 2, statistically significant differences (*p* = 0.037) were shown for a reduction of superficial EMG measurements differences (%) of the experimental group (DDN) with respect to intervention group (ICT) at a speed of 1 m/s (V1) immediately after both interventions, although not at speeds of 1.5 m/s (V2) or 2.5 m/s (V3).

### 3.3. Reliability Analysis

V1 EMG measurements showed excellent reliability with an ICC value of 0.969 (lower and upper limits of the 95% CI ranging from 0.946 to 0.984), SEM of 2.61% and MDC of 3.19%, both expressed in percentage with respect to the MVC. V2 EMG measurements also showed excellent reliability with an ICC value of 0.957 (lower and upper limits of the 95% CI ranging from 0.924 to 0.977), SEM of 3.07% and MDC of 3.46%, both expressed in percentage with respect to the MVC. V3 EMG measurements showed excellent reliability with an ICC value of 0.952 (lower and upper limits of the 95% CI from 0.914 to 0.975), SEM of 3.32% and MDC of 3.60%, both expressed in percentage with respect to the MVC. 

### 3.4. Multivariate Prediction Analysis

A statistically significant linear regression prediction model was shown for EMG outcome measurement differences at V1 (speed of 1 m/s) which was only predicted for the treatment group (*R*^2^ = 0.129; β = 8.054; F [1,32] = 4.734; *p* = 0.037) showing a reduction of this difference under DDN treatment. The rest of descriptive data, such as sex, height, weight, BMI and foot length, were excluded from this linear regression model according to the pre-stablished P_in_ = 0.05 and P_out_ = 0.10 values. Thus, descriptive data did not influence nor predict the EMG outcome measurement differences at V1 (speed of 1 m/s). In addition, linear regression models for EMG outcomes measurements differences at V2 (speed of 1.5 m/s) and V3 (speed of 2.5 m/sg) did not show any valid prediction model.

## 4. Discussion

DDN is an invasive treatment procedure that requires a thorough understanding of human anatomy in order to be properly performed [56]. It contains a risk of skin infection as a continuity solution occurs with this technique. Several cases of possible infection are described [57] and even Lee et al. described the development of an acute cervical epidural hematoma as a result of needling therapy in the area [43]. Therefore, a thorough knowledge of the anatomy is suggested to try to avoid these complications and to be thorough with hygiene measures. Treatment of latent MTrPs with DDN in gastrocnemius is related to intramuscular oedema, produced by the needling [50], procedure was associated with acute pain postneedling [50].

DDN of latent MTrPs leads to a temporary increase in muscle tone in the needling area, possibly due to intramuscular oedema in this area [58]. Regarding adverse effects of DDN, in 2014, Cummings et al. reported [59] a case of pneumothorax complications after DDN in the iliocostalis muscle. A deep spine infection [60] and infected of hip prothesis were also described too [57]. In addition, a cervical epidural hematoma [61] has been reported after DDN treatment. 

Common adverse effects included bruising, bleeding and pain during and after treatment. Correct technique, proper hygiene and anatomical knowledge are stipulated as preventive measures to avoid risks [62].

In case of obtaining similar results, how it happens in our study in the post treatment at 1.5 and 2.5 m/s speeds, with both techniques and taking into account the possible adverse effects resulting from DDN treatment [57,58,61], we would choose the ICT technique as the better treatment option in patients with latent MTrPs in gastrocnemius considering their EMG activity.

In the triceps surae the existence of latent MTrPs is associated with future muscular dysfunction [63]. 

A 2013 study [64], found a high percentage of latent MTrPs in the asymptomatic population and located a high prevalence of this point in the gastrocnemius muscle [64]

In keeping with the integrated hypothesis of a trigger point proposed by Simons, the zone around an MTrP is in an ischemic state with a shortage of glucose and oxygen [65]. Therefore, treatment with compression of MTrPs helps to improve sarcomere contractions [66].

Takamoto et al. demonstrated that compression applied to the MTrPs would affect to autonomic nervous activity [67]. They published a study in 2009 [67] in which MTrP compression elevated the activity of the parasympathetic nervous system and showed that the physiological mechanisms of pain relief could be induced by the pressure application over MTrPs. That increase entailed an increase in peripheral blood [68], one of the more important factors involved in relieving muscle fatigue [67]. The increased blood flow, provides a concurrent increased in the glucose availability to the muscle [69].

The insertion of a needle at the endplate region reduced the quantity of acetylcholine (ACh) by increasing discharges, leading to a lesser SEA. Hsieh et al. they found an increase in a number of hypoxic-responsive proteins after DDN stimulation that can promote angiogenesis, vasodilation, and altered glucose metabolism in MTrP location [69].

In this study, the effects of DDN at the precise site of latent MTrPs, as compared with those of ICT in the same location shows measure differences in the superficial EMG results, immediately after treatment, at 1 m/s speed. 

At a speed of 1 m/s, triathletes with latent MTPs, treated with DDN, immediately improve their muscular activity compared to those treated with ICT; in such a way that those triathletes who begin their recovery at low training speeds after injuries, they should receive as treatment, DDN. 

Based on our results, from 1.5 m/s of training speed, the activity data of muscle in triceps surae of triathletes with latent MTPs are similar, so treatment with ICT is recommended, when seeking to demonstrate less adverse effects.

A study on intramuscular EMG in latent MTrPs, in this case the trapezius, associated their existence with accelerated muscle fatigue, although patients did not have painful symptoms [65]. These results did not match what we found in our study, in which it was shown that despite deactivating the latent MTrP from both treatment techniques the results of EMG measurements were very similar to those before the treatment, perhaps conditioned by the immediately measurement.

With intramuscular EMG it is evident that a reduction in the fatigue progression measurement appears earlier in latent MTrPs muscle fibres than in muscle fibres without latent MTrP during a sustained isometric contraction [26]. These results suggest that latent MTrPs are associated with accelerated muscle fatigue.

The muscles fibres of latent MTrPs showed increased motor activity during contraction [10] when the measured was made with surface EMG. Measured with intramuscular EMG the activity in latent MTrPs was much higher than in areas where there is not latent MTrPs both at rest and in isometric contraction [24]. On the other hand when the measurement is made with surface EMG, there are no notable differences between the results obtained at rest and during the contraction [24].

Hong et al. conduced a study in 2014 in which an increase in surface EMG activity in latent MTrPs in the upper trapezius muscle when the muscle was submitted to low loads (25% of MVC) and short times (less than 10 s) of isometric contraction was not observed. On the other hand when the muscle was subject to low loads (25% of MVC) and somewhat longer times (7 min) of isometric and fatigue contractions an increase in surface EMG activity in latent MTrPs was induced [24]. These results may not contradict with those in our study because the performed contraction by triathletes was concentric in the Hong study. In 2019, Baraja-Vegas et al. published a study in which they used DN over latent MTrPs of medial gastrocnemius muscle and observed that the RMS peak amplitude of each subsequent LTR decreased when compared with the initial RMS peak amplitude of previous LTRs [70].

Another study in computer workers related to upper trapezius muscle demonstrated a small decrease in superficial EMG activity after the application of ICT [52] or DN [71]. 

In previous studies by our research group, we observed that both interventions DDN and ICT showed similarly efficacy in treatment of latent MTrPs in the gastrocnemius of triathletes in terms of dorsiflexion of tibiofibular-talar joint in addiction to changes in static and dynamic plantar pressures [36]. In addition, when our research group measured the pressure pain threshold (PPT) and thermography in latent MTrPs of triathletes treated with both techniques, we observed that local mechanosensitivity had immediately increased after treatment with DDN while this increase did not occur when triathletes were treated with ICT [72].

ICT has demonstrated moderately strong evidence for immediate pain relief in MTrPs, but this evidence is limited in terms of long-term pain relief. A recent review of the literature demonstrated moderately strong evidence supporting the use of ischemic pressure for immediate pain relief at the TrPs, but only limited evidence for long-term pain relief [73].

The great pressure stimulus caused by the needle on the MTrP sends strong neural impulses to the posterior horn, breaking the pain-spasm-pain circle of the MTrP as described by the gate control theory [74].

Hsieh et al. [75] demonstrated that when LTRs were elicited by DDN to a MTrP region suppression of spontaneous electrical activity (SEA) occurred.

According to the various theories, deactivation of trigger points may be attributed to mechanical [32], and biochemical [76] changes around needle insertion. One study demonstrated a decrease in motor end-plate hyperactivity in MTrP, in patients treated with DDN [77].

Sympathetic system regulation can be affected by the effects of needle insertion, and DDN might cause reduction of sympathetic response after treatment with this method [61].

Several authors have theorized about the possibility that rapid movement of the needle into a MTrP might stimulate afferent fibres and could block the pain information generated in the MTrP´s nociceptors though a ”gate control” mechanism [78].

In 2019, Barajas et al, conducted a study in which only superficial EMG changes, after the completion of DDN in latent MTrP were described. The decrease in local twitch response amplitudes (brief and sudden contractions of the MTrP taut band) peak after DDN with respect to before DDN treatment in the latent MTrPs [70]. 

In addition, treatment of latent MTrPs with DDN is related to intramuscular oedema, an improvement of muscle contraction reaction, and increase in muscle stiffness [57]. 

## 5. Limitations

As a main limitation, this study reflected only the EMG measurement results immediately after an intervention, and an intervention group with placebo treatment was not used.

Another limitation was pain measurements of were not obtained in this study because the aim in this study was measure EMG activity in latent MTrPs.

The fact that a single treatment session was carried out, and in the case of ICT, of only 60 s., could determine that the treatment was scarce in order to see results.

## 6. Conclusions

DDN administration requires experience and excellent anatomical knowledge. According to our findings immediately after treatment of latent MTrPs when the muscle was subdued due to a concentric contraction, DDN could be advisable for triathletes who train at a speed lower than 1 m/s, those who begin their recovery at low training speeds after injuries, they should receive as treatment, DDN, while ICT could be a more advisable technique than DDN for training or competition at speeds greater than 1.5 m/s. Further studies with longer follow-up periods and placebo interventions are suggested. 

## Figures and Tables

**Table 1:** Sociodemographic characteristics according to the division by treatment groups.

	Experimental Group (DDN) (*n* = 17)	Control Group (ICT) (*n* = 17)	*p* Value
Age (years)	35.29 ± 5.39 (32.73–37.85)	33.76 ± 5.76 (31.02–36.50)	0.215
Weight (kg)	65.17 ± 10.71(60.08–70.27)	69.17 ± 10.66 (64.10–74.24)	0.141
Height (cm)	170.35 ± 12.94 (164.19–176.50)	174.94 ± 6.96 (171.62–178.25)	0.103
BMI (kg/m^2^)	22.37 ± 1.92 (21.46–23.29)	22.48 ± 2.35 (21.36–23.6)	0.443
Foot length (cm)	41.55 ± 3.26 (40.00–43.11)	41.35 ± 2.73 (40.05–42.65)	0.421

Abbreviations; DDN, deep dry needling; IC, ischemic compression; m^2^, meter^2^; cm, centimetres; kg, kilograms; BMI, body mass index; 95% CI, confidence interval at 95%. Statistical significance for a *p* ˂ 0.05 value, with a confidence interval of 95%.

**Table 2:** Electromyographic measurement of muscle activity in medial or lateral gastrocnemius.

	Before Treatment			After Treatment			
Variable	Experimental Group (DDN)	Intervention Group (ICT)	*p* Value	Experimental Group (DDN)	Intervention Group (ICT)	Mean Difference (95% CI)	*p* Value
EMG FPM (µV). V1 (%)	33.75 ± 14.84 (26.69–40.81)	26.79 ± 9.40 (22.32–31.26)	0.056 **	−4.46 ± 11.53 (−30.74–11.08)	3.59 ± 10.00 (−9.29–30.33)	−8.05 (−15.60; −0.51)	0.037 * (*t* = −2.176)
EMG FPM (µV). V2 (%)	34.84 ± 14.43 (27.98–41.70)	30.36 ± 11.34 (24.97–35.76)	0.160 **	−1.00 ± 9.63 (−16.56–19.67)	4.45 ± 10.62 (−13.86–35.56)	−5.45 (−12.53; 1.63)	0.127 * (*t* = −1.167)
EMG FPM (µV). V3 (%)	38.69 ± 15.19 (31.46–45.91)	34.68 ± 11.69 (29.12–40.24)	0.197 **	2.13 ± 7.42 (−8.07–24.15)	3.23 ± 11.46 (−22.83–31.29)	−1.10 (−7.85; 5.84)	0.734 † (*U* = 155.00)

Abbreviations: DDN, deep dry needling; ICT, ischemic compression technique; FPM, mean power frequency (µV), % percentage with respect to the MVC; EMG, electromyography; V1, speed of 1 m/sg; V2, speed of 1.5 m/sg; V3, speed 2.5 m/sg; * Student’s parametric t test for independent samples. † Non-parametric Mann–Whitney U test for independent samples. Statistical significance for a *p*-value ˂ 0.05 (bold).

## Data Availability

Raw data will be available upon corresponding author request.
